# Behavioral Modulation of Infestation by *Varroa destructor* in Bee Colonies. Implications for Colony Stability

**DOI:** 10.1371/journal.pone.0160465

**Published:** 2016-09-01

**Authors:** Joyce de Figueiró Santos, Flávio Codeço Coelho, Pierre-Alexandre Bliman

**Affiliations:** 1 Applied Mathematics School, Getulio Vargas Foundation, Rio de Janeiro, RJ, Brasil; 2 Sorbonne Universités, Inria, UPMC Univ Paris 06, Lab. J.-L. Lions UMR CNRS 7598, Paris, France; University of North Carolina at Greensboro, UNITED STATES

## Abstract

Colony Collapse Disorder (CCD) has become a global problem for beekeepers and for the crops that depend on bee pollination. While many factors are known to increase the risk of colony collapse, the ectoparasitic mite *Varroa destructor* is considered to be the most serious one. Although this mite is unlikely to cause the collapse of hives itself, it is the vector for many viral diseases which are among the likely causes for Colony Collapse Disorder. The effects of *V. destructor* infestation differ from one part of the world to another, with greater morbidity and higher colony losses in European honey bees (EHB) in Europe, Asia and North America. Although this mite has been present in Brazil for many years, there have been no reports of colony losses amongst Africanized Honey Bees (AHB). Studies carried out in Mexico have highlighted different behavioral responses by the AHB to the presence of the mite, notably as far as grooming and hygienic behavior are concerned. Could these explain why the AHB are less susceptible to Colony Collapse Disorder? In order to answer this question, we have developed a mathematical model of the infestation dynamics to analyze the role of resistance behavior by bees in the overall health of the colony, and as a consequence, its ability to face epidemiological challenges.

## Introduction

In winter and spring of 2006/2007 American beekeepers started reporting heavier and widespread losses of bee colonies and so did Europeans beekeepers. This mysterious phenomenon was called “Colony Collapse Disorder” (CCD). Diseases, parasites, in-hive chemicals, agricultural insecticides, genetically modified crops, changed cultural practices and cool brood have all been suggested as possible causes for it [1] but nowadays the ectoparasitic mite *Varroa destructor* that parasitizes honey bees is considered the most likely cause. Although *V. destructor* has become a global problem its effects vary in different parts of the world. More intense losses have been reported in European honey bee colonies (EHB) in Europe, Asia and North America [2].

The mite’s life cycle is tightly linked with that of the bees. Immature mites develop with immature bees, parasitizing them from an early stage. The mite’s egg-laying behavior is coupled with that of the bees and thus depends on its reproductive cycle. In the northern hemisphere bees are much less active during the cold winter months. But since worker brood rearing (and thus *Varroa* reproduction) occurs all year round in tropical climates, one would expect that the impact of the parasite would be even worse in tropical regions. But even though *V. destructor* has been present in Brazil for more than 30 years, no colony collapses due to this mite, have been recorded [3]. It is worth noting that the dominant variety of bees in Brazil is the Africanized Honey Bee (AHB) which has spread throughout the entire country since its introduction in 1956 [4]. African bees and their hybrids are known to be more resistant to the mite *V. destructor* than the European bee subspecies [4, 5]. A review by Arechavaleta-Velasco et al. [6] in Mexico showed that EHB were twice as attractive to *V. destructor* as AHB.

### Resistance behaviors of the bee against the parasite

Both varieties of bees exhibit two types of resistance to the mite: firstly, grooming where workers use their legs and mandibles to remove the mite and then injure or kill it [7], and secondly hygienic behavior where workers destroy potentially infested brood cells [8]. Grooming behavior performed by adult bees, includes detecting and eliminating mites from their own body (auto-grooming) or from the body of another bee (allo-grooming). Hygienic behavior occurs when adult bees detect the presence of mite offspring still in the cells and in order to prevent the mites from spreading in the colony, the worker bees kill the infested brood. It has been demonstrated that the smell of the mite is capable of activating this behavior [9]. Hygienic behavior serves to combat other illnesses and parasites to which the brood is susceptible but it is not 100% accurate. Correa-Marques and De Jong [9] report that the majority (53%) of the uncapped cells display no apparent signs of parasitism or other abnormality which would justify killing of the brood.

AHB workers were more efficient in grooming mites from their bodies than EHB. AHB have been shown to be more effective in hygienic behavior than EHB. Vandame et al. [7] found in Mexico that the EHB are only able to remove 8% of infested brood whereas AHB removed up 32.5%. These types of behavior are important factors in keeping mite infestation low in the honey bee colonies but they come at a cost to the bees.

Our paper is not the first to model host-parasite systems; others exist in the literature and have recently been reviewed by Becher et al. [10]. In particular, Ratti et al. [11] modelled the population dynamics of bees and mites together with the acute bee paralysis virus. Here, we focus solely on the host-parasite interactions in order to understand the resilience of colonies in Brazil and the role of the more efficient resistance behaviors displayed by AHB to explain the lower infestation rates and the lower incidence of colony collapse [7].

The main goal of this paper is to propose a model capable of describing the dynamics of infestation by *V. destructor* in bee colonies taking into consideration bee’s resistance mechanisms to mite infestation, grooming and hygienic behavior. In addition, by simulating the dynamics, we show how the resistance behaviors contribute to reducing infestation levels in the colony.

## Mathematical model

Vandame et al. [12] discuss the cost-benefit of resistance mechanism of bee against mite. The *grooming* behavior performed by adult bees, includes detecting and eliminating mites from their own body (auto-grooming) or from the body of another bee (allo-grooming). The hygienic behavior occurs when adult bees detect the presence of the mite offspring still in the cells and in order to prevent the mites from spreading in the colony, the worker bees kill the infested brood. Their study compared the results for two subspecies of bees—Africanized and European—to examine whether these two mechanisms could explain the observed low compatibility between Africanized bees and the mite *Varroa destructor*, in Mexico. The results showed that *grooming* and hygienic behavior appears most intense in Africanized bees than in Europeans bees.

**Fig 1:**
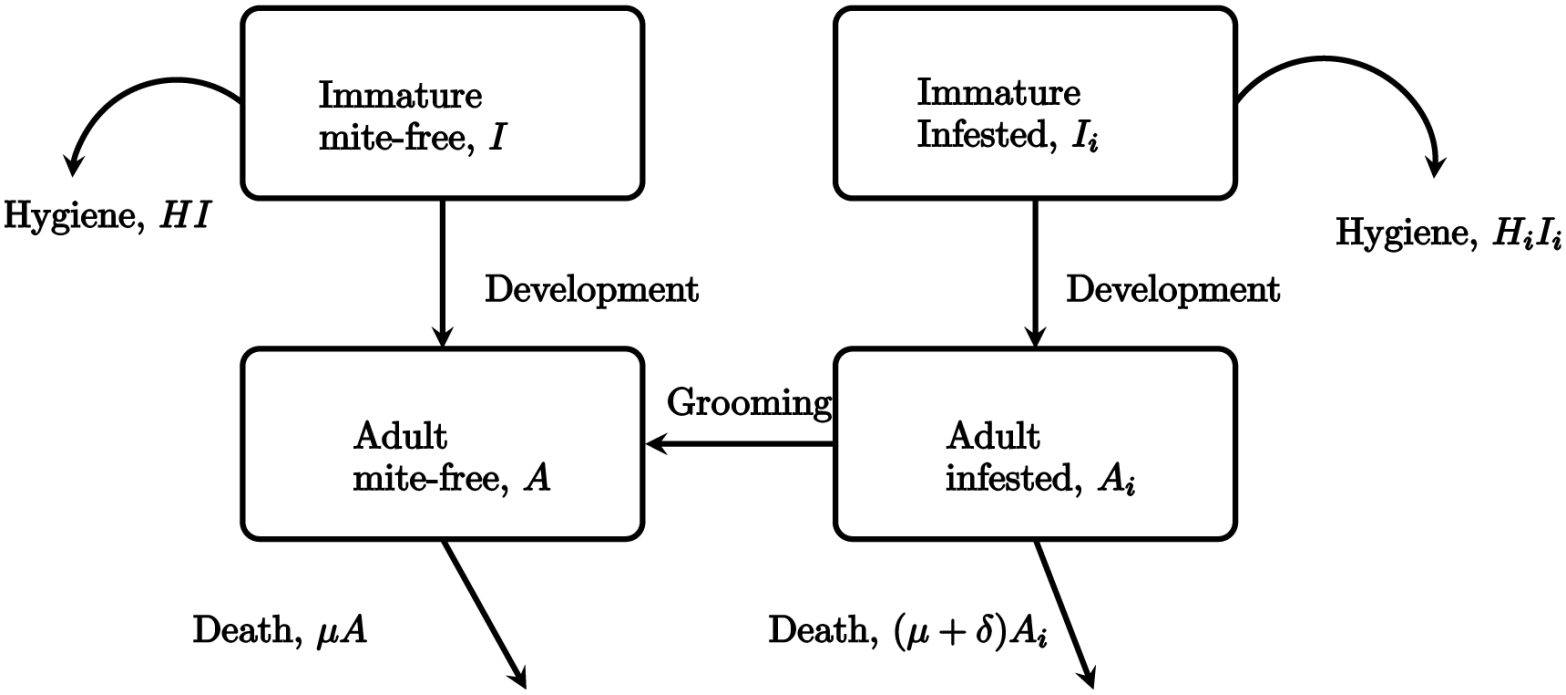
Diagram to describes the dynamics of the model.

The model proposed is shown in the diagram of Fig 1, and detailed in the system of differential equations below:

$$\begin{array}{ccc} \dot{I} & = & \pi \frac{A}{A+A_{i}}-\delta I-HI\\ \dot{A} & = & \delta I+gA_{i}-\mu A\\ \dot{I_{i}} & = & \pi \frac{A_{i}}{A+A_{i}}-\delta I_{i}-H_{i}I_{i}\\ \dot{A_{i}} & = & \delta I_{i}-gA_{i}-\left(\mu +\gamma \right)A_{i} \end{array}$$ (1)

In the proposed model, *I*, *I*_*i*_, *A* and *A*_*i*_ represent the non-infested immature bees, infested immature bees, non-infested adult worker bees and infested adult worker bees, respectively.

Daily birth rate for bees is denoted by *π*, *δ* is the maturation rate, i.e., the inverse of number of days an immature bee requires to turn in adult, this rate is the same for both infested and non-infested immature bees. The infestation of immature bees is proportional to the fraction of infested adults because females mites initiate reproduction by entering the brood cell, before it is sealed [2]. *μ* is the mortality rate for adult bees, *γ* is the mortality rate induced by the presence of mites in the colony bees. The value used for *γ* (Table 1) is insignificant, but this parameter can be used in extensions of this model to represent additional mortality due to the impact of diseases transmitted by the mite. The parameters *H*_*i*_, *H* and *g* are the rate of removal of infested pupae via hygienic behavior, the general hygienic rate (affecting uninfested pupae) and grooming rate, respectively.

**Table 1:** Parameters of the model.

Parameters	Meaning	Value	Unit	Reference
*π*	Bee daily birth rate	2500	*bees* × *day*^−1^	[13]
*δ*	Maturation rate	0.05	*day*^−1^	[13]
*H*	Generic hygienic behavior	-	*day*^−1^	-
*H*_*i*_	Hygienic behavior towards infested brood	-	*day*^−1^	-
*g*	*Grooming*	-	*day*^−1^	-
*μ*	Mortality rate	0.04	*day*^−1^	[14]
*γ*	Mite induced mortality	10^−7^	*day*^−1^	[11]

### Choosing parameters values

Some of the parameters associated with the bees life cycle, used for the simulations, can be found in the literature, as shown in Table 1. For the resistance behavior parameters, *g*, *H* and *H*_*i*_, very little information is available. Therefore we decided to study the variation of these parameters within ranges which allowed for the system to switch between a mite-free equilibrium to one of stable infestation. These ranges also reflected observations described in the literature (Table 2) [6, 12, 15].

**Table 2:** Varying the parameters.

Parameter	Minimum value	Maximum value
*g*	0.01	0.01
*H*_*i*_	0.08	0.4
*H*	0.04	0.2

The three unknown parameters representing resistance behaviors *g*, *H*_*i*_, *H*—grooming, proper hygienic behavior and harmful hygienic behavior—were studied with respect to the existence of a stable infestation equilibrium.

## Results

### Basic reproduction number $R_{0}$ of the infested bees

One way of looking for a boundary beyond which infestation by mites is possible, is to compute the basic reproduction number, $R_{0}$ of infestation. For our model, the basic reproduction number, or $R_{0}$ of infested bees, can be thought of as the number of new infestations that one infested bee when introduced into the colony generates on average over the course of its infestation period or before it is groomed, in an otherwise uninfested population.

#### Deriving $R_{0}$ using the next-generation method

To calculate the basic reproduction number of infested bees, we will use the next-generation matrix [16], where the whole population is divided into *n* compartments in which there are *m* < *n* infested compartments. The next-generation matrix defines the instantaneous rate of expansion of the infestation, right at the start.

In this method, $R_{0}$ is defined as the spectral radius, or the largest eigenvalue, of the next-generation matrix.

Let *x*_*i*_, *i* = 1, 2, …, *m* be the number or proportion of individuals in the *ith* compartment. Then

$$\begin{array}{c} \frac{dx_{i}}{dt}=F_{i}\left(x\right)-V_{i}\left(x\right) \end{array}$$

where $F_{i}\left(x\right)$ is the rate of appearance of new infestations in compartment *i* and $V_{i}\left(x\right)=V_{i}^{-}\left(x\right)-V_{i}^{+}\left(x\right)$. $V_{i}^{-}$ is the rate of transfer of individuals out of the *ith* compartment, and $V_{i}^{+}$ represents the rate of transfer of individuals into compartment *i* by all other means.

The next-generation matrix is then defined by *FV*^−1^, where *F* and *V* can be formed by the partial derivatives of Fi and $V_{i}$.

$$\begin{array}{c} F=\left[\frac{\partial F_{i}\left(x_{0}\right)}{\partial x_{j}}\right]\;\text{and}\;V=\left[\frac{\partial V_{i}\left(x_{0}\right)}{\partial x_{j}}\right] \end{array}$$

where *x*_0_ is the disease free equilibrium.

In our model, *m* = 2 and the infested compartments are:

$$\begin{array}{ccc} \frac{dI_{i}}{dt} & = & \pi \frac{A_{i}}{A+A_{i}}-\delta I_{i}-H_{i}I_{i}\\ \frac{dA_{i}}{dt} & = & \delta I_{i}-gA_{i}-\left(\mu +\gamma \right)A_{i} \end{array}$$ (2)

Now we write the matrices F and V, substituting the mite-free equilibrium values, $A^{*}=\frac{\delta \pi }{\mu (\delta +H)}$ and $A_{i}^{*}=0$.

$$\begin{array}{c} F=[\begin{array}{cc} 0 & \frac{\mu (\delta +H}{\delta })\\ 0 & 0 \end{array}] \end{array}$$

$$\begin{array}{c} V=[\begin{array}{cc} \delta +H_{i} & 0\\ -\delta & g+\gamma +\mu \end{array}] \end{array}$$

Let the next-generation matrix *G* be the matrix product *FV*^−1^. Then

$$\begin{array}{c} G=[\begin{array}{cc} \frac{\mu (\delta +H)}{\left(\delta +H_{i}\right)\left(g+\gamma +\mu \right)} & \frac{\mu (\delta +H)}{\delta (g+\gamma +\mu )}\\ 0 & 0 \end{array}] \end{array}$$

Now we can find the basic reproduction number, $R_{0}$, which is the largest eigenvalue of the matrix *G*.

$$\begin{array}{c} R_{0}=\frac{\mu (\delta +H)}{\left(\delta +H_{i}\right)\left(g+\gamma +\mu \right)} \end{array}$$ (3)

**Fig 2:**
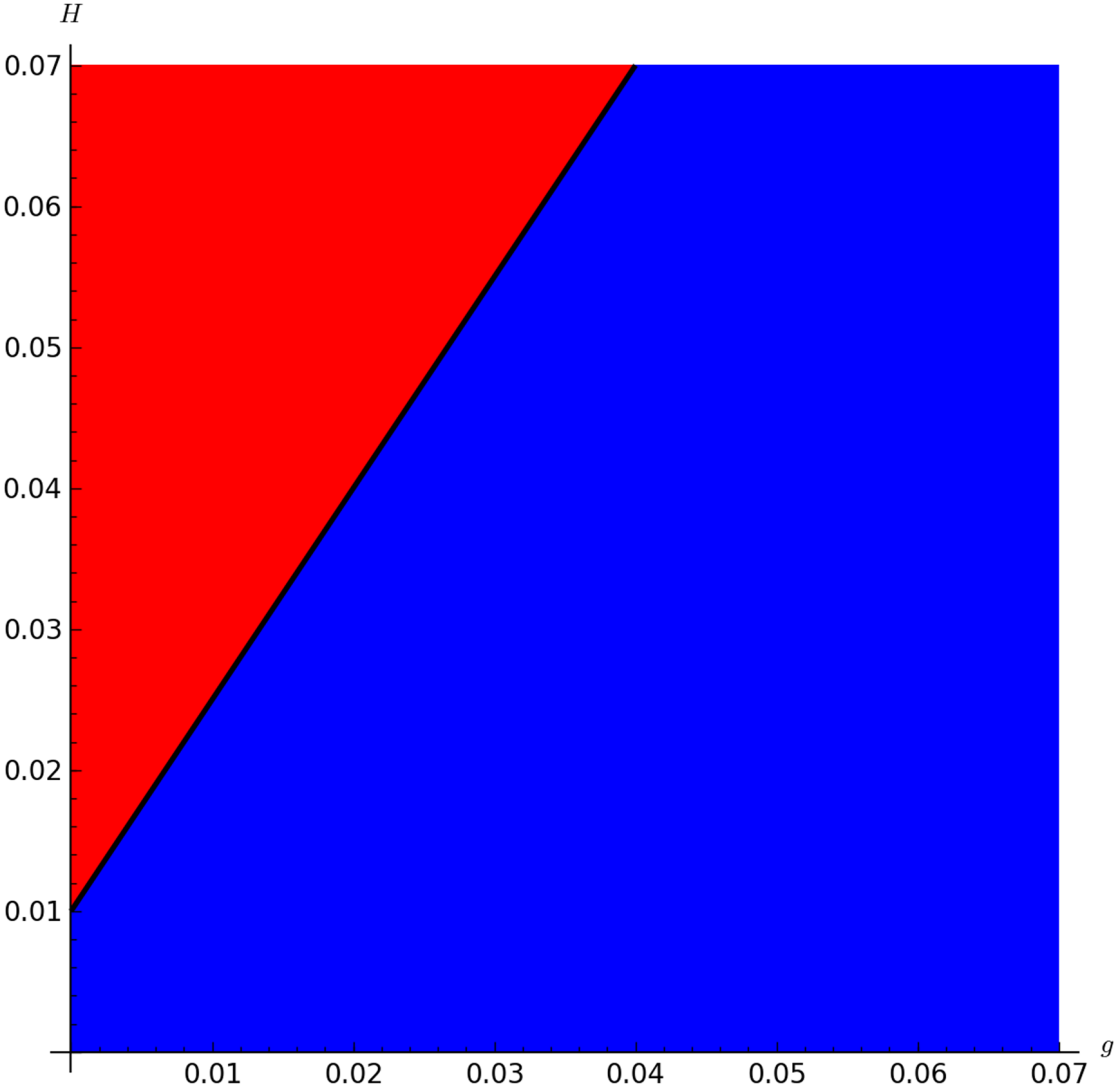
Plot of values of $R_{0}$ for a range of values of *g* and *H*. *H*_*i*_ = 0.01 and remaining parameters set as described in Table 1. The region in red (top-left) corresponds to $R_{0}>1$, the black line to $R_{0}=0$ and the blue region (bottom-right) otherwise. This figure shows a slightly narrower range of the parameters as described in Table 2, for a better visualization of the threshold.

**Fig 3:**
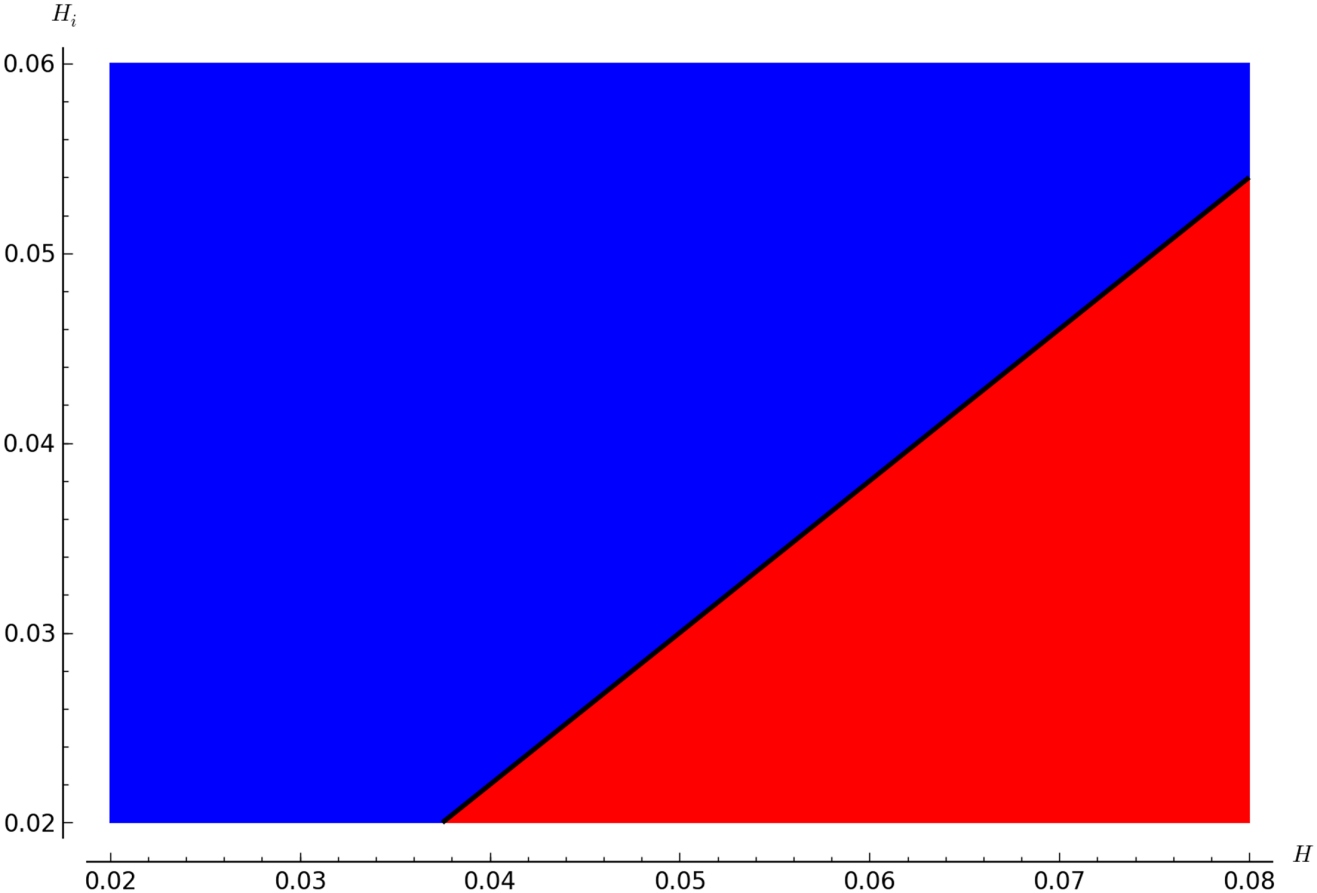
Values of $R_{0}$ for various combinations of *H*_*i*_ and *H*. *g* = 0.01 and other parameters as given in Table 1. The region in red (bottom-right) corresponds to $R_{0}>1$, the black line to $R_{0}=0$ and the blue region (top-left) otherwise. This figure illustrates one of the conditions for infestation(given other parameters values fixed as in Table 1) that *H* must be larger than *H*_*i*_. This figure shows a slightly narrower range of the parameters as described in Table 2, for a better visualization of the threshold.

**Fig 4:**
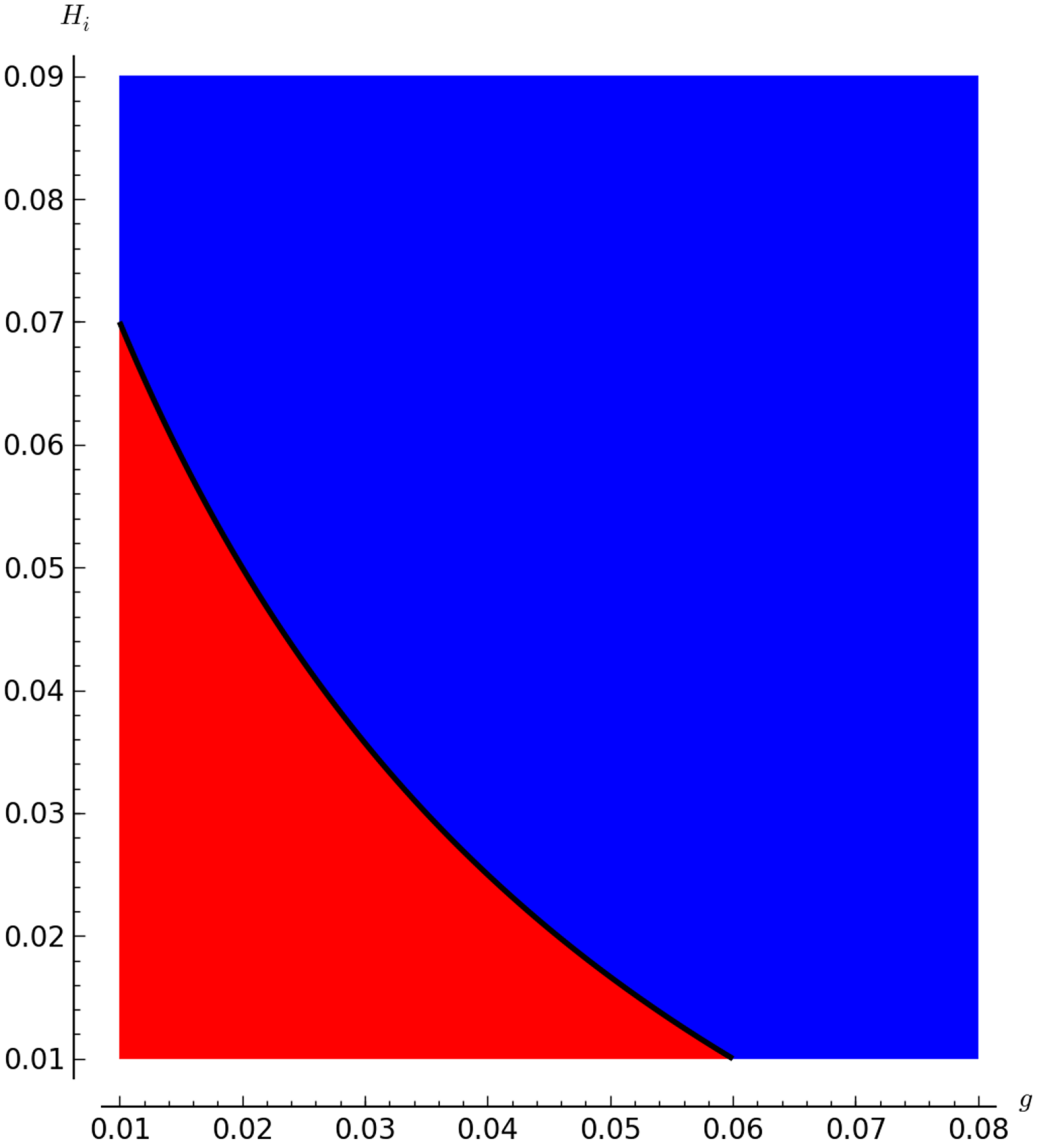
Implicit plot for $R_{0}$ letting *g* and *H*_*i*_ vary. Using the values for parameters *π*, *δ*, *μ*, *H* and *γ* from Table 1 The red region represent $R_{0}>1$ which means that for these combination of *g* and *H*_*i*_ the mite will stay in the colony. On the other hand, the blue region represents $R_{0}<1$ which means that for these these combination of *g* and *H*_*i*_ the mites will be eliminated.

Figs 2, 3 and 4 show the boundary between mite-free (blue region, $R_{0}<1$) and infestation equilibria (red region, $R_{0}>1$).

### Well-Posed and Boundedness

For sake of simplicity, we denote

$$\begin{array}{c} \alpha ≐\delta +H,\;\alpha_{i}≐\delta +H_{i},\;\mu_{i}≐\mu +\gamma \end{array}$$ (4)

in such a way that the System (1) rewrites

$$\begin{array}{c} \dot{I}=\pi \frac{A}{A+A_{i}}-\alpha I \end{array}$$ (5a)

$$\begin{array}{c} \dot{A}=\delta I-\mu A+gA_{i} \end{array}$$ (5b)

$$\begin{array}{c} {\dot{I}}_{i}=\pi \frac{A_{i}}{A+A_{i}}-\alpha_{i}I_{i} \end{array}$$ (5c)

$$\begin{array}{c} {\dot{A}}_{i}=\delta I_{i}-\left(\mu_{i}+g\right)A_{i} \end{array}$$ (5d)

We assume that all the coefficients presented in Table 1 are all positive, that is:

$$\begin{array}{c} \pi ,\delta ,\mu >0,\;\alpha ,\alpha_{i}>\delta ,\;\mu_{i}>\mu . \end{array}$$ (6)

The previous system of equations is written

$$\begin{array}{c} \dot{X}=f\left(X\right),\;X=\left(I,A,I_{i},A_{i}\right) \end{array}$$ (7)

The right-hand side of Eq (7) is not properly defined in the points where *A* + *A*_*i*_ = 0. However, the following result demonstrates that this has no consequence on the solutions, as the latter stays away from this part of the subspace. For subsequent use, we denote D the subset of those elements $X=\left(I,A,I_{i},A_{i}\right)\in R_{+}^{4}$ such that *A* + *A*_*i*_ ≠ 0.

**Theorem 1** (Well-posedness and boundedness) *If* $X_{0}\in D$, *then there exists a unique solution of* Eq (7) *defined on* [0, +∞) *such that* *X*(0) = *X*_0_. *Moreover, for any* *t* > 0, X(t)∈D, *and*

$$\begin{array}{c} \frac{\pi }{\alpha_{\max}}\leq \underset{t\to +\infty }{\text{lim inf}}\left(I\left(t\right)+I_{i}\left(t\right)\right)\leq \underset{t\to +\infty }{\text{lim sup}}\left(I\left(t\right)+I_{i}\left(t\right)\right)\leq \frac{\pi }{\alpha_{\min}} \end{array}$$ (8a)

$$\begin{array}{c} \frac{\delta \pi }{\mu_{i}\alpha_{\max}}\leq \underset{t\to +\infty }{\text{lim inf}}\left(A\left(t\right)+A_{i}\left(t\right)\right)\leq \underset{t\to +\infty }{\text{lim sup}}\left(A\left(t\right)+A_{i}\left(t\right)\right)\leq \frac{\delta \pi }{\mu \alpha_{\min}} \end{array}$$ (8b)

*where by definition* *α*_min_ ≐ min{*α*; *α*_*i*_}, *α*_max_ ≐ max{*α*; *α*_*i*_}. *Also*,

$$\begin{array}{c} \frac{1}{(\alpha -\alpha_{\min})\mu +\alpha g}\frac{\pi g\mu \alpha_{\min}}{\mu_{i}\alpha_{\max}}\leq \underset{t\to +\infty }{\text{lim inf}}I\left(t\right),\;\frac{1}{(\alpha -\alpha_{\min})\mu +\alpha g}\frac{\delta \pi g\alpha }{\mu_{i}\alpha_{\max}}\leq \underset{t\to +\infty }{\text{lim inf}}A\left(t\right) \end{array}$$ (9)

*and*

$$\begin{array}{c} \left(I_{i}\left(0\right),A_{i}\left(0\right)\right)\neq \left(0,0\right)\Rightarrow \forall t\geq 0,\;I_{i}\left(t\right)>0,\;A_{i}\left(t\right)>0 \end{array}$$ (10)

Define $D^{\prime }$ as the largest set included in D and fulfilling the inequalities of Theorem 1, that is:

$$\begin{array}{c} D^{\prime }≐\{\left(I,A,I_{i},A_{i}\right)\in R_{+}^{4}:\frac{\pi g\mu \alpha_{\min}}{\mu_{i}\alpha_{\max}}\leq I,\;\frac{\delta \pi g\alpha }{\mu_{i}\alpha_{\max}}\leq A,\;\frac{\pi }{\alpha_{\max}}\leq I+I_{i}\leq \frac{\pi }{\alpha_{\min}},\\ \frac{\delta \pi }{\mu_{i}\alpha_{\max}}\leq A+A_{i}\leq \frac{\delta \pi }{\mu \alpha_{\min}}\}. \end{array}$$ (11)

Theorem 1 shows that the compact set $D^{\prime }$ is positively invariant and attracts all the trajectories. Therefore, in order to study the asymptotics of System (5), it is sufficient to consider the trajectories of Eq (5) that are in $D^{\prime }$.

In Theorem 1, the notations lim inf and lim sup correspond respectively to the limit inferior and limit superior of a function (or lower limit and upper limit). We recall e.g. that the limit superior at infinity of a real-valued function *f* defined on [0, +∞) is equal to inf_*t* ≥ 0_sup_*τ* ≥ *t*_ *f*(*τ*). It is the largest accumulation point of *f* at infinity.

### Equilibria

**Theorem 2** (Equilibria and asymptotic behavior) *Define*

$$\begin{array}{c} \beta ≐\frac{\mu }{\alpha_{i}}-\frac{\mu_{i}+g}{\alpha } \end{array}$$ (12)

• *If* *β* ≤ 0, *then there exists a unique equilibrium point of* System (7) *in* $D^{\prime }$, *that corresponds to a mite-free situation. It is globally asymptotically stable, and given by*

$$\begin{array}{c} X_{MF}=\frac{\pi }{\alpha }(\begin{array}{c} 1\\ \frac{\delta }{\mu }\\ 0\\ 0 \end{array}). \end{array}$$ (13)

• *If* $\beta >\frac{1}{\alpha_{i}}$, *then there exists two equilibrium points in* $D^{\prime }$, *namely* *X*_*MF*_ *and a infestation equilibrium defined by*

$$\begin{array}{c} X_{CO}=\frac{\delta \pi g}{\alpha_{i}\left(\mu_{i}+g\right)}\frac{\alpha \mu -\alpha_{i}\left(\mu_{i}+g\right)}{\alpha \left(\mu +g\right)-\alpha_{i}\left(\mu_{i}+g\right)}(\begin{array}{c} \frac{1}{\delta }\frac{\alpha_{i}\left(\mu_{i}+g\right)}{\alpha \mu -\alpha_{i}\left(\mu_{i}+g\right)}\\ \frac{\alpha }{\alpha \mu -\alpha_{i}\left(\mu_{i}+g\right)}\\ \frac{\mu_{i}+g}{\delta g}\\ \frac{1}{g} \end{array}). \end{array}$$ (14)

*Moreover, for all initial conditions in* $D^{\prime }$ *except in a zero measure set, the trajectories tend towards* *X*_*CO*_.

Recall that $R_{0}=\frac{\alpha \mu }{\alpha_{i}\left(\mu_{i}+g\right)}$, in such a way that

$$\begin{array}{c} \beta >0\Leftrightarrow R_{0}>1. \end{array}$$ (15)

**Fig 5:**
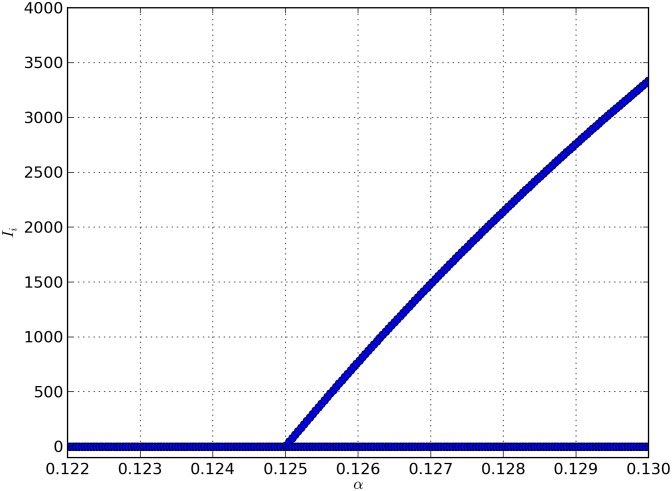
Bifurcation diagram showing the transcritical bifurcation with bifurcation point *α*_0_ ≈ 0.125 (*β* = 0, $R_{0}=1$). When the parameter *α* is greater than *α*_0_, coexistence equilibrium (*I*_*i*_ > 0) exists. When *α* < *α*_0_, only the mite-free equilibrium exists. Blue dots correspond to the equilibrium values of *I*_*i*_.

The point $R_{0}=1$, that is *β* = 0, is the point of a transcritical bifurcation, that appears when $R_{0}$ gets larger than 1. For larger values, two equilibria are found analytically, a mite-free one, that is unstable, and a infestation equilibrium which is stable. We’ve shown (Theorem 2) that the latter is globally asymptotically stable if $\beta >\frac{1}{\alpha_{i}}$, and conjecture that the same property holds for *β* in the interval $\left(0,\frac{1}{\alpha_{i}}\right]$. Using *α* as bifurcation parameter, the bifurcation appears for $\alpha =\frac{\alpha_{i}\left(\mu_{i}+g\right)}{\mu }\approx 0.125$, after substituting the parameter values (Fig 5).

**Fig 6:**
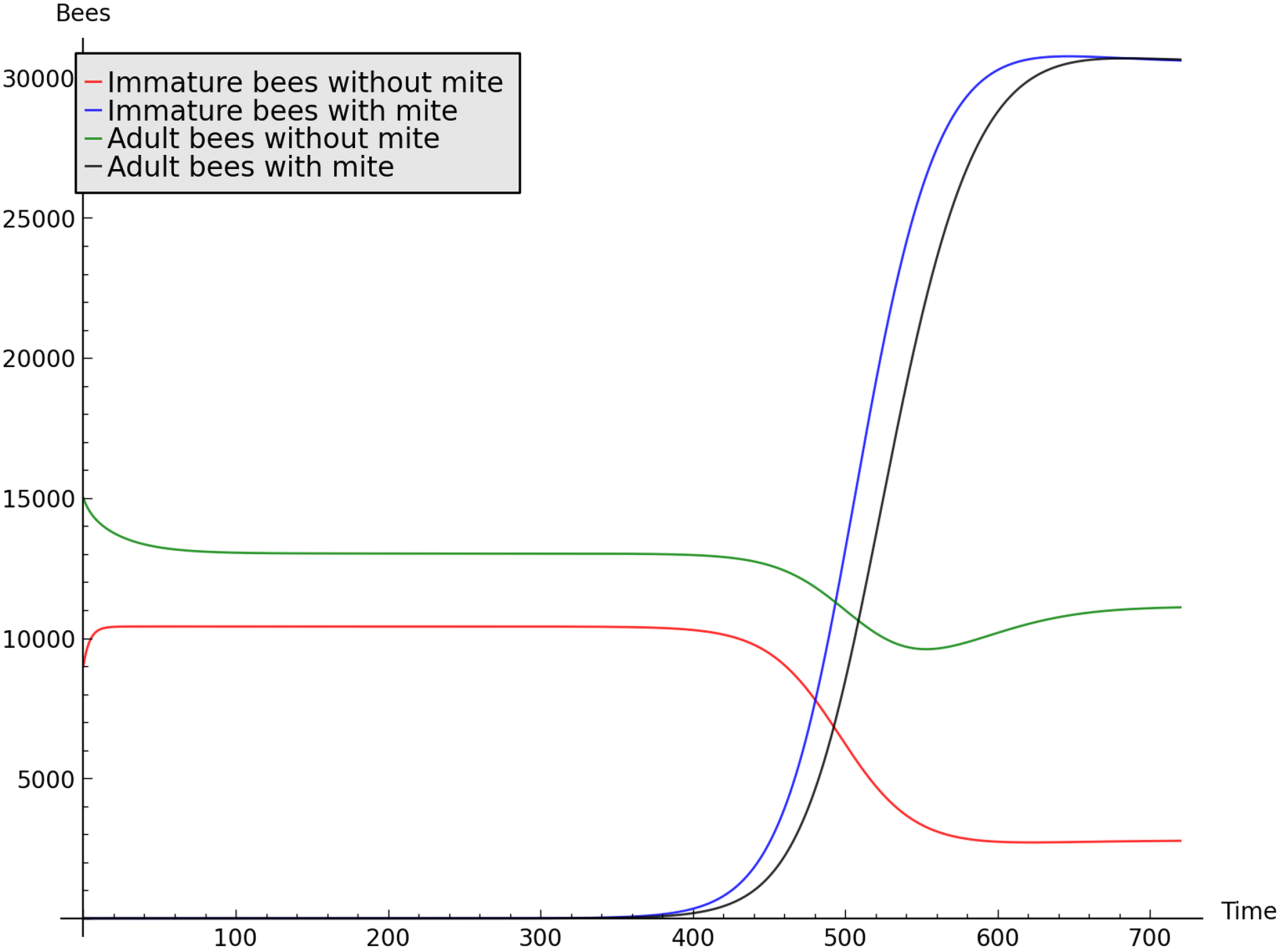
Simulation showing the infestation of a colony, by a single infested adult bee, with parameters giving $R_{0}\approx 1.33$. Initial conditions: *I* = 5000, *I*_*i*_ = 0, *A* = 20000, *A*_*i*_ = 0 and parameters *g* = 0.01, *H*_*i*_ = 0.1, *μ* = 0.04, *δ* = 0.05, *γ* = 10^−7^ and *H* = 0.19. On time *t* = 100 days, a single infested adult bee is introduced into the colony. For this simulation, *β* = 0.375 and $R_{0}\approx 3.199$.

If we solve numerically the system from Eq (5), we confirm the existence of two equilibria when *α* crosses the bifurcation value of 0.125. The instability and stability of the mite-free and infestation equilibria, respectively is shown in the simulation of Fig 6.

**Fig 7:**
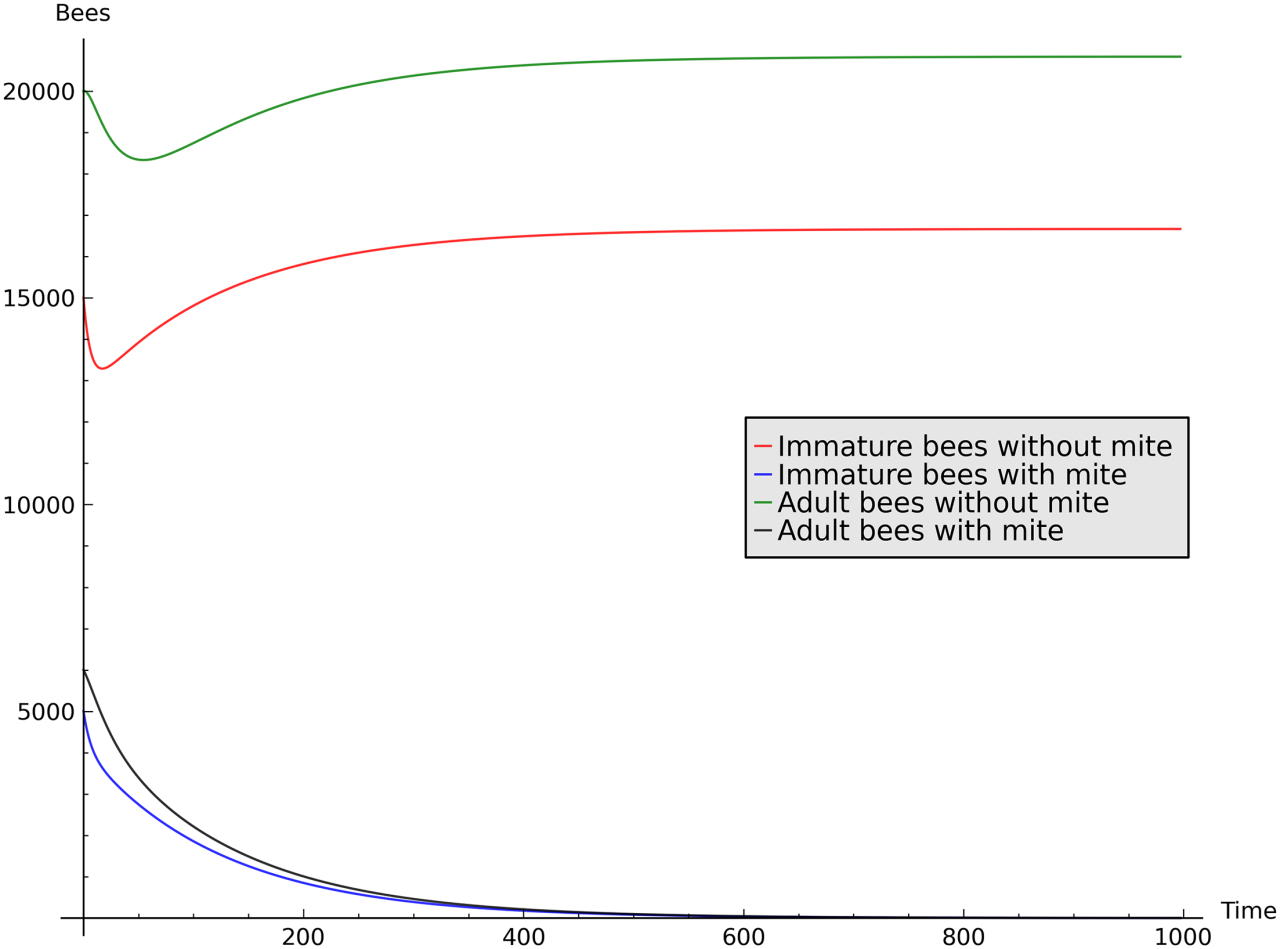
Simulation showing the elimination of the mites from a colony, by a single infested adult bee, when *R*_0_ < 1. Initial conditions: *I* = 15000, *I*_*i*_ = 5000, *A* = 20000, *A*_*i*_ = 6000 and parameters *g* = 0.01, *H*_*i*_ = 0.1, *μ* = 0.05, *δ* = 0.05, *γ* = 10^−7^ and *H* = 0.1.

Figs 6 and 7 show simulations representing the infestation and mite-free equilibria, respectively. The time range of simulations is between 2 and 3 years, with daily time steps, which is enough for the dynamic to converge to the equilibria.

## Proofs of the theorems

**Proof of Theorem 1**. • Clearly, the right-hand side of the system of equations is globally Lipschitz on any subset of D where *A* + *A*_*i*_ is bounded away from zero. The existence and uniqueness of the solution of System (5) is then obtained for each trajectory staying at finite distance of this boundary. We will show that the two formulas provided in the statement are valid for each trajectory departing initially from a point where *A* + *A*_*i*_ ≠ 0. As a consequence, the fact that all trajectories are defined on infinite horizon will ensue.

• Summing up the first two equations in Eq (5) yields, for any point inside D:

$$\begin{array}{c} \dot{I}+{\dot{I}}_{i}=\pi -\alpha I-\alpha_{i}I_{i}\geq \pi -\alpha_{\max}\left(I+I_{i}\right). \end{array}$$ (16)

Integrating this differential inequality between any two points *X*(0) = *X*_0_ and *X*(*t*) of a trajectory for which X(τ)∈D, *τ* ∈ [0; *t*], one gets

$$\begin{array}{c} I\left(t\right)+I_{i}\left(t\right)\geq \frac{\pi }{\alpha_{\max}}(1-e^{-\alpha_{\max}t})+\left(I\left(0\right)+I_{i}\left(0\right)\right)e^{-\alpha_{\max}t}, \end{array}$$ (17)

where the right-hand side is in any case positive for any *t* > 0.

Similarly, one has

$$\begin{array}{c} \dot{I}+{\dot{I}}_{i}\leq \pi -\alpha_{\min}\left(I+I_{i}\right), \end{array}$$ (18)

and therefore

$$\begin{array}{c} I\left(t\right)+I_{i}\left(t\right)\leq \frac{\pi }{\alpha_{\min}}(1-e^{-\alpha_{\min}t})+\left(I\left(0\right)+I_{i}\left(0\right)\right)e^{-\alpha_{\min}t}. \end{array}$$ (19)

This proves in particular that the inequalities in Eq (8a) hold for any portion of trajectory remaining inside D.

We now consider the evolution of *A*, *A*_*i*_. Similarly to what was done for *I*, *I*_*i*_, one has

$$\begin{array}{c} \dot{A}+{\dot{A}}_{i}=\delta \left(I+I_{i}\right)-\mu A-\mu_{i}A_{i}\geq \delta \left(I+I_{i}\right)-\mu_{i}\left(A+A_{i}\right) \end{array}$$ (20)

Therefore,

$$\begin{array}{c} A\left(t\right)+A_{i}\left(t\right)\geq \left(A\left(0\right)+A_{i}\left(0\right)\right)e^{-\mu_{i}t}+\delta \int_{0}^{t}\left(I\left(\tau \right)+I_{i}\left(\tau \right)\right)e^{-\mu_{i}\left(t-\tau \right)}.\;d\tau . \end{array}$$ (21)

Integrating the lower bound of *I* + *I*_*i*_ extracted from Eq (17) yields the conclusion that any solution departing from D indeed remains in D as long as it is defined. On the other hand, we saw previously that trajectories remaining in D could be extended on the whole semi-axis [0, +∞). Therefore, any trajectory departing from a point in D can be extended to [0, +∞), and remains in D for any *t* > 0. In particular, Eq (8a) holds for any trajectory departing inside D.

Let us now achieve the proof by bounding *A* + *A*_*i*_ from above. One has

$$\begin{array}{c} \dot{A}+{\dot{A}}_{i}\leq \delta \left(I+I_{i}\right)-\mu \left(A+A_{i}\right) \end{array}$$ (22)

and thus

$$\begin{array}{c} A\left(t\right)+A_{i}\left(t\right)\leq \left(A\left(0\right)+A_{i}\left(0\right)\right)e^{-\mu t}+\delta \int_{0}^{t}\left(I\left(\tau \right)+I_{i}\left(\tau \right)\right)e^{-\mu (t-\tau )}.\;d\tau . \end{array}$$ (23)

Using Eq (19) then permits to achieve the proof of Eq (8b), and finally the proof of Eq (8).

• Let us now prove Eq (9). One deduces from Eqs (5a) and (5b) and the bounds established earlier the differential inequalities

$$\begin{array}{c} \dot{I}\geq \frac{\pi }{\text{lim}\;\text{sup}(A+A_{i})}A-\alpha I\geq \frac{\mu \alpha_{\min}}{\delta }A-\alpha I, \end{array}$$ (24a)

$$\begin{array}{c} \dot{A}\geq \delta I-\mu A+g\left(\text{lim}\;\text{inf}\left(A+A_{i}\right)-A\right)\geq \delta I-\left(\mu +g\right)A+\frac{\delta \pi g}{\mu_{i}\alpha_{\max}} \end{array}$$ (24b)

The auxiliary linear time-invariant system

$$\begin{array}{c} \frac{d}{dt}(\begin{array}{c} I^{\prime }\\ A^{\prime } \end{array})=(\begin{array}{cc} -\alpha & \frac{\mu \alpha_{\min}}{\delta }\\ \delta & -(\mu +g) \end{array})(\begin{array}{c} I^{\prime }\\ A^{\prime } \end{array})+(\begin{array}{c} 0\\ \frac{\delta \pi g}{\mu_{i}\alpha_{\max}} \end{array}) \end{array}$$ (25)

is monotone, as the state matrix involved is a Metzler matrix [17]. Moreover, it is asymptotically stable, as the associated characteristic polynomial is equal to

$$\begin{array}{c} |\begin{array}{cc} s+\alpha & -\frac{\mu \alpha_{\min}}{\delta }\\ -\delta & s+\mu +g \end{array}|=s^{2}+\left(\alpha +\mu +g\right)s+\alpha \left(\mu +g\right)-\mu \alpha_{\min}, \end{array}$$ (26)

and thus Hurwitz because *α*(*μ* + *g*) − *μα*_min_ = (*α* − *α*_min_)*μ* + *αg* > 0. Therefore, all trajectories of Eq (25) tend towards the unique equilibrium:

$$\begin{array}{ccc} \lim_{t\to +\infty }(\begin{array}{c} I^{\prime }\left(t\right)\\ A^{\prime }\left(t\right) \end{array}) & = & -{\left(\begin{array}{cc} -\alpha & \frac{\mu \alpha_{\min}}{\delta }\\ \delta & -(\mu +g) \end{array}\right)}^{-1}(\begin{array}{c} 0\\ \frac{\delta \pi g}{\mu_{i}\alpha_{\max}} \end{array})\\ & = & \frac{1}{(\alpha -\alpha_{\min})\mu +\alpha g}(\begin{array}{cc} \mu +g & \frac{\mu \alpha_{\min}}{\delta }\\ \delta & \alpha \end{array})(\begin{array}{c} 0\\ \frac{\delta \pi g}{\mu_{i}\alpha_{\max}} \end{array})\\ & = & \frac{1}{(\alpha -\alpha_{\min})\mu +\alpha g}(\begin{array}{c} \frac{\pi g\mu \alpha_{\min}}{\mu_{i}\alpha_{\max}}\\ \frac{\delta \pi g\alpha }{\mu_{i}\alpha_{\max}} \end{array}). \end{array}$$ (27)

Invoking Kamke’s Theorem, see e.g. ([18] Theorem 10, p. 29), one deduces from Eq (24) and the monotony of Eq (25) the following comparison result, that holds for all trajectories of Eq (31):

$$\begin{array}{c} \underset{t\to +\infty }{\text{lim inf}}(\begin{array}{c} I(t)\\ A(t) \end{array})\geq \frac{1}{(\alpha -\alpha_{\min})\mu +\alpha g}(\begin{array}{c} \frac{\pi g\mu \alpha_{\min}}{\mu_{i}\alpha_{\max}}\\ \frac{\delta \pi g\alpha }{\mu_{i}\alpha_{\max}} \end{array}). \end{array}$$ (28)

This gives Eq (9).

• One finally proves Eq (10). Using Eq (8b), identity Eq (5c) implies

$$\begin{array}{c} {\dot{I}}_{i}\geq \frac{\pi }{\text{lim}\;\text{sup}(A+A_{i})}A_{i}-\alpha_{i}I_{i}\geq \frac{\mu \alpha_{\min}}{\delta }A_{i}-\alpha_{i}I_{i} \end{array}$$ (29)

Joining this with Eq (5d) and using Kamke’s result as before, ones deduces that both *I*_*i*_ and *A*_*i*_ have positive values when at least one of their two initial values are positive. This achieves the proof of Theorem 1.

**Proof of Theorem 2**. The proof is organized as follows.

1. We first write System (5) under the form of an I/O system, namely
  $$\begin{array}{c} \dot{I}=\pi \frac{A}{A+A_{i}}-\alpha I \end{array}$$ (30a)
  $$\begin{array}{c} \dot{A}=\delta I-\mu A+u \end{array}$$ (30b)
  $$\begin{array}{c} {\dot{I}}_{i}=\pi \frac{A_{i}}{A+A_{i}}-\alpha_{i}I_{i} \end{array}$$ (30c)
  $$\begin{array}{c} {\dot{A}}_{i}=\delta I_{i}-\left(\mu_{i}+g\right)A_{i} \end{array}$$ (30d)
  $$\begin{array}{c} y=gA_{i} \end{array}$$ (30e)
  where *u*, resp. *y*, is the input, resp. the output, closed by the unitary feedback
  $$\begin{array}{c} u=y. \end{array}$$ (30f)
  For subsequent use of the theory of monotone systems, one determines, for any (nonnegative) constant value of *u*, the equilibrium values of (*I*, *A*, *I*_*i*_, *A*_*i*_) for Eqs (30a)–(30d), and the corresponding values of *y* as given by Eq (30e).
2. The equilibrium points of System (5) are then exactly (and easily) obtained by solving the fixed point problem *u* = *y* among the solutions of the previous problem.
  unique equilibrium points when *β* ≤ 0, and there exist exactly two equilibrium points when *β* > 0. equilibrium points.
3. One then shows that the I/O system *u* ↦ *y* defined by Eqs (30a)–(30e) is anti-monotone with respect to certain order relation, and the study of the stability of these equilibria shows that it admits single-valued I/S and I/O characteristics, as in [19].
4. Using this properties, the stability of the equilibria of the system obtained by closing the loop Eqs (30a)–(30e) by Eq (30f) is then established using arguments similar to Angeli and Sontag [17].

1. For fixed *u* > 0, the equilibrium equations of the I/O system (30a)–(30e) are given by

$$\begin{array}{c} \pi \frac{A}{A+A_{i}}-\alpha I=0 \end{array}$$ (31a)

$$\begin{array}{c} \delta I-\mu A+u=0 \end{array}$$ (31b)

$$\begin{array}{c} \pi \frac{A_{i}}{A+A_{i}}-\alpha_{i}I_{i}=0 \end{array}$$ (31c)

$$\begin{array}{c} \delta I_{i}-\left(\mu_{i}+g\right)A_{i}=0 \end{array}$$ (31d)

$$\begin{array}{c} y=gA_{i} \end{array}$$ (31e)

Summing up the first and third identities gives

$$\begin{array}{c} \pi =\alpha I+\alpha_{i}I_{i}, \end{array}$$ (32)

and thus necessarily:

$$\begin{array}{c} \exists \lambda \in \left[0;1\right],\;I=\lambda \frac{\pi }{\alpha },\;I_{i}=\left(1-\lambda \right)\frac{\pi }{\alpha_{i}}. \end{array}$$ (33)

• The case *λ* = 0 yields *I* = 0, and then *A* = 0 by Eq (31a), and therefore *u* has to be zero from Eq (31b). Also, $I_{i}=\frac{\pi }{\alpha_{i}}$, $A_{i}=\frac{\delta \pi }{\alpha_{i}\left(\mu_{i}+g\right)}$ by Eq (31d), and then $y=gA_{i}=\frac{g\delta \pi }{\alpha_{i}\left(\mu_{i}+g\right)}$. in Eq (11) and should be discarded. obtained point is located outside D and has to be discarded; or

• The case *λ* = 1 yields *I*_*i*_ = 0, and then *A*_*i*_ = 0 by Eqs (31d) or (31c), and *y* = 0. There remains the two following conditions:

$$\begin{array}{c} \pi =\alpha I,\;\delta I=\mu A-u \end{array}$$ (34)

which yield

$$\begin{array}{c} I=\frac{\pi }{\alpha },\;A=\frac{\delta \pi }{\alpha \mu }+\frac{u}{\mu } \end{array}$$ (35)

(The map *u* ↦ *y*(*u*) is therefore multivalued.) Notice that these solutions do *not* systematically correspond to equilibrium points for the closed-loop System (30). unconditionally.

• Let us now look for possible values of *λ* in (0;1). From Eqs (33) and (31a)–(31c), one deduces

$$\begin{array}{c} \frac{A}{A_{i}}=\frac{\alpha I}{\alpha_{i}I_{i}}=\frac{\lambda }{1-\lambda }. \end{array}$$ (36)

Using Eq (33) on the one hand and summing the two identities Eqs (31b)–(31d) on the other hand, yields

$$\begin{array}{c} \delta \left(I+I_{i}\right)=\delta \pi (\frac{\lambda }{\alpha }+\frac{1-\lambda }{\alpha_{i}})=\mu A+\left(\mu_{i}+g\right)A_{i}-u=A(\mu +\left(\mu_{i}+g\right)\frac{1-\lambda }{\lambda })-u. \end{array}$$ (37)

This permits to express *A* as a function of *λ*, namely:

$$\begin{array}{c} A=\frac{\lambda }{\lambda \mu +\left(1-\lambda \right)\left(\mu_{i}+g\right)}[\delta \pi (\frac{\lambda }{\alpha }+\frac{1-\lambda }{\alpha_{i}})+u]. \end{array}$$ (38)

Using this formula together with Eqs (33), (31d) and (36) now allows to find an equation involving only the unknown *λ*, namely:

$$\begin{array}{ccc} \delta I_{i}=\frac{\delta \pi }{\alpha_{i}}\left(1-\lambda \right) & = & \left(\mu_{i}+g\right)A_{i}=\left(\mu_{i}+g\right)\frac{A_{i}}{A}A\\ & = & \left(\mu_{i}+g\right)\frac{1-\lambda }{\lambda }\frac{\lambda }{\lambda \mu +\left(1-\lambda \right)\left(\mu_{i}+g\right)}[\delta \pi (\frac{\lambda }{\alpha }+\frac{1-\lambda }{\alpha_{i}})+u]. \end{array}$$ (39)

Simplifying (as *λ* ≠ 0, 1) gives:

$$\begin{array}{c} \frac{\delta \pi }{\alpha_{i}}=\frac{\mu_{i}+g}{\lambda \mu +\left(1-\lambda \right)\left(\mu_{i}+g\right)}[\delta \pi (\frac{\lambda }{\alpha }+\frac{1-\lambda }{\alpha_{i}})+u]. \end{array}$$ (40)

The previous condition is clearly affine in *λ*. It writes

$$\begin{array}{c} \left(\lambda \mu +\left(1-\lambda \right)\left(\mu_{i}+g\right))\frac{\delta \pi }{\alpha_{i}}=\left(\mu_{i}+g\right)(\delta \pi (\frac{\lambda }{\alpha }+\frac{1-\lambda }{\alpha_{i}})+u\right) \end{array}$$ (41)

which, after developing and simplifying, can be expressed as:

$$\begin{array}{c} \lambda \mu \frac{\delta \pi }{\alpha_{i}}=\left(\mu_{i}+g\right)(\delta \pi \frac{\lambda }{\alpha }+u) \end{array}$$ (42)

and finally

$$\begin{array}{c} \left(\mu_{i}+g\right)u=\delta \pi (\frac{\mu }{\alpha_{i}}-\frac{\mu_{i}+g}{\alpha })\lambda =\delta \pi \beta \lambda . \end{array}$$ (43)

For *u* ≥ 0, this equation admits a solution in (0;1) if and only if

$$\begin{array}{c} \beta >0\;\;\text{and}\;\;u<u^{*}≐\frac{\delta \pi \beta }{\mu_{i}+g}, \end{array}$$ (44)

and the latter is given as

$$\begin{array}{c} \lambda =\frac{\mu_{i}+g}{\delta \pi \beta }u. \end{array}$$ (45)

The state and output values may then be expressed explicitly as functions of *u*. In particular, one has

$$\begin{array}{c} y\left(u\right)=gA_{i}=\frac{\delta g}{\mu_{i}+g}I_{i}=\frac{\delta \pi g}{\alpha_{i}\left(\mu_{i}+g\right)}\left(1-\lambda \right)=\frac{\delta \pi g}{\alpha_{i}\left(\mu_{i}+g\right)}(1-\frac{\mu_{i}+g}{\delta \pi \beta }u). \end{array}$$ (46)

value

**Fig 8:**
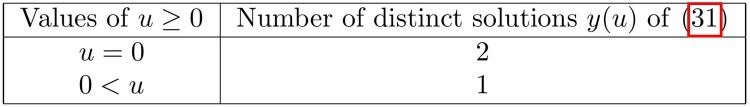
$R_{0}\leq 1$ (i.e. *β* ≤ 0). See details in the text.

**Fig 9:**
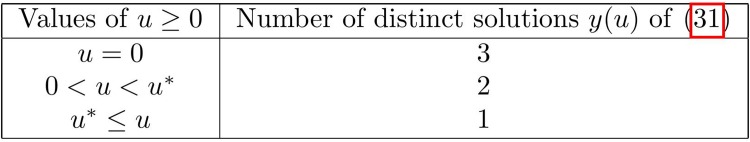
$R_{0}>1$ (i.e. *β* > 0). See details in the text.

•Eq (31) admits exactly one solution in $D^{\prime }$ for any *u* ≥ 0; admits a supplementary solution in $D^{\prime }$ for any *u* ∈ [0; u*). Figs 8 and 9 summarize the number of solutions of Eq (31) for all nonnegative values of *u*. (The map *u* ↦ *y*(*u*) is therefore multivalued.) Notice that these solutions do *not* systematically correspond to equilibrium points for the closed-loop System (30).

2. The equilibrium points of System (5) are exactly those points for which *u* = *y*(*u*) for some nonnegative scalar *u*, where *y*(*u*) is one of the output values corresponding to *u* previously computed. We now examine in more details the solutions of this equation.

• For the value *λ* = 0 in the previous computations, one should have *u* = 0, due to Eq (45); but on the other hand *y* > 0 for *u* = 0, due to Eq (46). Therefore this point does not correspond to an equilibrium point of System (31).

• The value *λ* = 1 yields a unique equilibrium point. Indeed, *y* = 0, so *u* should be zero too, and the unique solution is given by

$$\begin{array}{c} I=\frac{\pi }{\alpha },\;A=\frac{\delta \pi }{\alpha \mu },\;I_{i}=0,\;A_{i}=0,\;y=0. \end{array}$$ (47)

This corresponds to the equilibrium denoted *X*_*MF*_ in the statement.

• Let us consider now the case of *λ* ∈ (0;1). For this case to be considered, it is necessary that *β* > 0, that is $R_{0}>1$. The value of *u* should be such that (see Eq (46))

$$\begin{array}{c} y=\frac{\delta \pi g}{\alpha_{i}\left(\mu_{i}+g\right)}-\frac{g}{\alpha_{i}\beta }u=u, \end{array}$$ (48)

that is

$$\begin{array}{c} (1+\frac{g}{\alpha_{i}\beta })u=\frac{\delta \pi g}{\alpha_{i}\left(\mu_{i}+g\right)}, \end{array}$$ (49)

or again

$$\begin{array}{c} u=\frac{\delta \pi \beta g}{\left(\alpha_{i}\beta +g\right)\left(\mu_{i}+g\right)}=\frac{\delta \pi g}{\alpha_{i}\left(\mu_{i}+g\right)}\frac{\alpha \mu -\alpha_{i}\left(\mu_{i}+g\right)}{\alpha \left(\mu +g\right)-\alpha_{i}\left(\mu_{i}+g\right)}, \end{array}$$ (50)

after replacing *β* by its value defined in Eq (12). The corresponding value of

$$\begin{array}{c} \lambda =\frac{\mu_{i}+g}{\delta \pi \beta }u=\frac{g}{\alpha_{i}\beta +g}, \end{array}$$ (51)

given by Eq (45), is clearly contained in (0;1) when *β* > 0. Therefore, when *β* > 0, there also exists a second equilibrium. The latter is given by:

$$\begin{array}{c} I=\lambda \frac{\pi }{\alpha }=\frac{\mu_{i}+g}{\alpha \delta \beta }u=\frac{1}{\delta }\frac{\alpha_{i}\left(\mu_{i}+g\right)}{\alpha \mu -\alpha_{i}\left(\mu_{i}+g\right)}u,\;A_{i}=\frac{u}{g}, \end{array}$$ (52a)

$$\begin{array}{c} I_{i}=\frac{\mu_{i}+g}{\delta }A_{i}=\frac{\mu_{i}+g}{\delta g}u \end{array}$$ (52b)

$$\begin{array}{c} A=\frac{1}{\mu }(\delta I+u)=\frac{1}{\mu }(\frac{\alpha_{i}\left(\mu_{i}+g\right)}{\alpha \mu -\alpha_{i}\left(\mu_{i}+g\right)}+1)u=\frac{\alpha }{\alpha \mu -\alpha_{i}\left(\mu_{i}+g\right)}u, \end{array}$$ (52c)

and corresponds to *X*_*CO*_ defined in the statement.

diagonal that comes from the loop closing.

3. Let K be the cone in $R_{+}^{4}$ defined as the product of orthants $R_{+}\times R_{+}\times R_{-}\times R_{-}$. We endow the state space with this order. In other words, for any *X* = (*I*, *A*, *I*_*i*_, *A*_*i*_) and $X^{\prime }=\left(I^{\prime },A^{\prime },I_{i}^{\prime },A_{i}^{\prime }\right)$ in $R_{+}^{4}$, $X\leq_{K}X^{\prime }$ means:

$$\begin{array}{c} I\leq I^{\prime },A\leq A^{\prime },I_{i}\geq I_{i}^{\prime },A_{i}\geq A_{i}^{\prime }. \end{array}$$ (53)

With this structure, one may verify that the System (30a)–(30e) has the following monotonicity properties [20, 21]

- For any function u∈U≐{u:[0;+∞)→R, locally integrable and taking on positive values almost everywhere}, for any $X_{0},X_{0}^{\prime }\in R_{+}^{4}$,
  $$\begin{array}{c} X_{0}\leq_{K}X_{0}^{\prime }\;\Rightarrow \;\forall t\geq 0,\;X\left(t;X_{0},u\right)\leq_{K}X\left(t;X_{0}^{\prime },u\right) \end{array}$$ (54)
  where by definition *X*(*t*; *X*_0_, *u*) denotes the value at time *t* of the point in the trajectory departing at time 0 from *X*_0_ and subject to input *u*.
- The Jacobian matrix of the I/O system is
  $$\begin{array}{c} (\begin{array}{cccc} -\alpha & \pi \frac{A_{i}}{{\left(A+A_{i}\right)}^{2}} & 0 & -\pi \frac{A}{{\left(A+A_{i}\right)}^{2}}\\ \delta & -\mu & 0 & 0\\ 0 & -\pi \frac{A_{i}}{{\left(A+A_{i}\right)}^{2}} & -\alpha_{i} & \pi \frac{A}{{\left(A+A_{i}\right)}^{2}}\\ 0 & 0 & \delta & -(\mu_{i}+g) \end{array}), \end{array}$$ (55)
  which is irreducible when *A* ≠ 0 and *A*_*i*_ ≠ 0. The system is therefore strongly monotone in $D^{\prime }\setminus \left\{X:A_{i}=0\right\}$ (notice that $D^{\prime }$ does not contain points for which *A* = 0), and also on the invariant subset $D^{\prime }\cap \left\{X:I_{i}=0,\;A_{i}=0,\right\}$.
- The input-to-state map is monotone, that is: for any inputs $u,u^{\prime }\in U$, for any $X_{0}\in R_{+}^{4}$,
  $$\begin{array}{c} u\left(t\right)\leq u^{\prime }\left(t\right)\;\text{a.e.}\;\Rightarrow \;\forall t\geq 0,\;X\left(t;X_{0},u\right)\leq_{K}X\left(t;X_{0}^{\prime },u\right). \end{array}$$ (56)
- The state-to-output map is anti-monotone, that is: for any $X,X^{\prime }\in R_{+}^{4}$,
  $$\begin{array}{c} X\leq_{K}X^{\prime }\;\Rightarrow \;\forall t\geq 0,\;gA_{i}\geq gA_{i}^{\prime } \end{array}$$ (57)

monotone (due to the irreducibility of the Jacobian matrix) for any constant value of *u*.

• In order to construct I/S and I/O characteristics for System (31), we now examine the stability of the equilibria of System (31) for any fixed value of $u\in R_{+}$. As shown by Theorem 1, all trajectories are precompact.

• When *β* ≤ 0, it has been previously established that for any u∈R there exists at most one equilibrium in $D^{\prime }$ to the I/O System (31). The strong monotonicity property of this system depicted above then implies that this equilibrium is globally attractive ([20] Theorem 10.3). Therefore, System (31) possesses I/S and I/O characteristics. As for any value of *u*, this equilibrium corresponds to zero output, the I/O characteristics is zero. Applying the results of Angeli and Sontag [19], one gets that the closed-loop system equilibrium *X*_*MF*_ is an almost globally attracting equilibrium for System (5).

• Let us now consider the case where *β* > 0. We first show that the equilibrium point with *I*_*i*_ = 0, *A*_*i*_ = 0 and Eq (34) is locally unstable. Notice that this point is located on a branch of solution parametrized by *u* and departing from *X*_*MF*_ for *u* = 0. The Jacobian matrix Eq (55) taken at this point is

$$\begin{array}{c} (\begin{array}{cccc} -\alpha & 0 & 0 & -\frac{\mu \alpha \pi }{\delta \pi +\alpha u}\\ \delta & -\mu & 0 & 0\\ 0 & 0 & -\alpha_{i} & \frac{\mu \alpha \pi }{\delta \pi +\alpha u}\\ 0 & 0 & \delta & -(\mu_{i}+g) \end{array}). \end{array}$$ (58)

This matrix is block triangular, with diagonal blocks

$$\begin{array}{c} (\begin{array}{cc} -\alpha & 0\\ \delta & -\mu \end{array})\;\;\text{and}\;\;(\begin{array}{cc} -\alpha_{i} & \frac{\mu \alpha \pi }{\delta \pi +\alpha u}\\ \delta & -(\mu_{i}+g) \end{array}). \end{array}$$ (59)

The first of them is clearly Hurwitz, while the second, whose characteristic polynomial is

$$\begin{array}{ccc} s^{2}+\left(\alpha_{i}+\mu_{i}+g\right)s+\alpha_{i}\left(\mu_{i}+g\right)-\frac{\mu \alpha \delta \pi }{\delta \pi +\alpha u} & = & s^{2}+\left(\alpha_{i}+\mu_{i}+g\right)s-\alpha \alpha_{i}\left(\beta -u\left(\mu_{i}+g\right)\right)\\ & = & s^{2}+\left(\alpha_{i}+\mu_{i}+g\right)s-\alpha \alpha_{i}\left(\mu_{i}+g\right)\left(u^{*}-u\right) \end{array}$$ (60)

(where u* is defined in Eq (44)) is not Hurwitz when *β* > 0 and 0 ≤ *u* ≤ u*, and has a positive root for 0 < *u* < u*. Therefore, the corresponding equilibrium of the I/O System (30) is unstable for these values of *u*.

The other solution, given as a function of *u* by Eq (52), is located on a branch of solution parametrized by *u* and departing from *X*_*CO*_ for *u* = 0. As the other solution is unstable for 0 < *u* < u*, one can deduce from Hirsch [20] that these solutions are locally asymptotically stable.

• One may now associate to any *u* ∈ [0; u*] the corresponding unique locally asymptotically stable equilibrium point, and the corresponding output value, defining therefore respectively an I/S characteristic *k*_*X*_ and an I/O characteristic *k* for System (30).

For any scalar *u* ∈ [0; u*], for almost any $X_{0}\in D^{\prime }$, one has

$$\begin{array}{c} \lim_{t\to +\infty }X\left(t;X_{0},u\right)=k_{X}\left(u\right),\;\lim_{t\to +\infty }y\left(t;X_{0},u\right)=k\left(u\right), \end{array}$$ (61)

and, from the monotony properties, for any scalar-valued continuous function *u*, for almost any $X_{0}\in D^{\prime }$:

$$\begin{array}{c} k(\underset{t\to +\infty }{\text{lim sup}}u\left(t\right))\leq \underset{t\to +\infty }{\text{lim inf}}y\left(t;X_{0},u\right)\leq \underset{t\to +\infty }{\text{lim sup}}y\left(t;X_{0},u\right)\leq k(\underset{t\to +\infty }{\text{lim inf}}u\left(t\right)). \end{array}$$ (62)

Using the fact that *k* is anti-monotone and that *u* = *y* for the closed-loop system, one deduces, as e.g. in Gouzé [22] that, for the solutions of the latter,

$$\begin{array}{c} k^{2l}(\underset{t\to +\infty }{\text{lim inf}}y\left(t;X_{0},u\right))\leq \underset{t\to +\infty }{\text{lim inf}}y\left(t;X_{0},u\right)\leq \underset{t\to +\infty }{\text{lim sup}}y\left(t;X_{0},u\right)\leq k^{2l}(\underset{t\to +\infty }{\text{lim sup}}y\left(t;X_{0},u\right)). \end{array}$$ (63)

Here *k*(*u*), defined by Eq (46), is a linear decreasing map. When its slope is smaller than 1, then the sequences in the left and right of Eq (63) tend towards the fixed point that corresponds to the output value at *X* = *X*_*CO*_, see Eq (50).

This slope value, see Eq (46), is equal to

$$\begin{array}{c} \frac{\delta \pi g}{\alpha_{i}\left(\mu_{i}+g\right)}\frac{\mu_{i}+g}{\delta \pi \beta }=\frac{1}{\alpha_{i}\beta }, \end{array}$$ (64)

and it thus smaller than 1 if and only if $\beta >\frac{1}{\alpha_{i}}$, which is an hypothesis of the statement.

Under these assumptions, one then obtains that the lim inf and lim sup in Eq (63) are equal, and thus that *y*, and thus *u*, possesses limit for *t* → +∞. Moreover, the state itself converges towards the equilibrium *X*_*CO*_ when *t* → +∞ for almost every initial conditions *X*(0). This achieves the proof of Theorem 2.

## Discussion

The parasitism of bees by Varroa mites in nature is an undeniable fact. However, this parasitic relationship is fraught with dangers for the bees, since Varroa mites can be vectors of lethal viral diseases. These deleterious effects for the health of the individual workers and the whole colony, has led to the evolution of resistance behaviors such as the hygienic behavior and grooming.

Those behaviors are not entirely without cost to the bees, exacerbated hygienic behavior—when both *H* and *H*_*i*_ are intensified—can exert a substantial toll on the fitness of the queen. So it is safe to say that this parasitic relationship has evolved within a very narrow range of parameters. Even if the mite-free equilibrium is advantageous to the colony, maintaining it may be too expensive to the bees.

On the other hand, in the absence of viral diseases, mite parasitism seems to be fairly harmless. If we look at the expression for the $R_{0}$ of infestation Eq (3), we can see that the mite-induced bee mortality, *γ*, must be kept low or risk de-stabilizing the colony.

Africanized Honey Bees, having evolved more effective resistance behaviors, are more resistant to colony colapse through this ability to keep infestation levels lower when compared to their European counterparts [23, 24]. Unfortunately, the lack of more detailed experiments measuring the rates of grooming and higienic behaviors in both groups (EHB and AHB), makes it hard to position them accurately in the parameter space of the model presented.

In this model, we chose to leave seasonal effects out, for simplicity, even though it is known that colonies in temperate climates suffer substantial losses during the winter. Such effects can be added to this model through the use of a time-varying mortality and birth rates. Nevertheless, we are convinced this simple model still applies to tropical colonies, and our observations about infestation levels and colony vulnerability remain relevant regardless of external morbidity factors such as hard winters.

Finally, we hope that the model presented here along with its demonstrated dynamical properties will serve as a solid foundation for the development of other models including viral dynamics and other aspects of bee colony health.
